# Extraction, Chemical Composition, and Anticancer Potential of *Origanum onites* L. Essential Oil

**DOI:** 10.3390/molecules24142612

**Published:** 2019-07-18

**Authors:** Katerina Spyridopoulou, Eleni Fitsiou, Eleni Bouloukosta, Angeliki Tiptiri-Kourpeti, Manolis Vamvakias, Antigoni Oreopoulou, Eleni Papavassilopoulou, Aglaia Pappa, Katerina Chlichlia

**Affiliations:** 1 Department of Molecular Biology and Genetics, Democritus University of Thrace, University Campus-Dragana, 68100 Alexandroupolis, Greece; 2 VIORYL S.A., Chemical & Agricultural Industry, Research S.A., 19014 Afidnes, Greece

**Keywords:** *Origanum onites*, essential oil, colon cancer, antitumor, oral administration

## Abstract

*Origanum* species are plants rich in volatile oils that are mainly used for culinary purposes. In recent years, there has been a growing interest in the biological activities of their essential oils. *Origanum onites* L. is a plant mainly found in Greece, Turkey, and Sicily, whose oil is rich in carvacrol, a highly bioactive phytochemical. The aim of this study was to analyze the chemical composition of *Origanum onites* essential oil (OOEO), and investigate its potential anticancer effects in vitro and in vivo. GC/MS analysis identified carvacrol as OOEO’s main constituent. In vitro antiproliferative activity was assayed with the sulforhodamine B (SRB) assay against human cancer cell lines from four tumor types. HT-29, a colorectal cancer cell line, was the most sensitive to the antiproliferative activity of OOEO. Wound-healing assay and Annexin V-PI staining were employed to investigate the antimigratory and the pro-apoptotic potential of OOEO, respectively, against human (HT-29) and murine (CT26) colon cancer cells. Notably, OOEO attenuated migration and induced apoptosis-related morphological changes in both cell lines. Prophylactic oral administration of the oil in a BALB/c experimental mouse model inhibited the growth of syngeneic CT26 colon tumors. As far as we know, this is the first report on the antitumor potential of orally administered OOEO.

## 1. Introduction

Plant-derived natural products have been used throughout human history in traditional medicine practices of various ethnic groups [1]. In later years, it became evident that the development of novel pharmaceuticals based on bioactive natural products would be defining the future of global healthcare [2,3,4]. Since the identification and the isolation of morphine, the first pharmacologically-active compound from the opium plant in 1805 [5], several bioactive substances have been identified, characterized, and isolated from various plants, highlighting that natural products lead the way to the discovery of new therapeutic compounds for the treatment of human diseases [6]. 

Specifically, meticulous research conducted over the past decades has led to the identification of numerous dietary plant-derived compounds with chemopreventive potential [7]. Volatile phytochemicals that constitute essential oils of various edible plants are among the dietary elements that have been shown to possess significant antitumor and chemopreventive activity against colon cancer [8].

*Origanum onites* L. (*O. onites*) is a plant of the genus *Origanum*. In general, *Origanum* species are rich in volatile oils comprising mainly carvacrol and thymol [9]. *O. onites* is primarily a culinary herb and is cultivated for its aromatic leaves. The plant’s commercial name is “Turkish oregano”. Its distribution area is limited in the southern part of Greece, a small region in Sicily and, as its commercial name implies, in southwestern Turkey [10]. 

The essential oil extracted from *O. onites* has stimulated scientific interest, as it has been shown to exert a diverse range of biological activities [11], such as antifungal, antimicrobial [12], antioxidant [13], insecticidal [14], hepatoprotective [15], and cytotoxic activity [16]. Moreover, various extracts of the plant have been reported to possess antiproliferative properties against cancer cells in vitro [17,18,19].

Therefore, the aim of this study was to extract the essential oil from the plant *O. onites*, analyze its chemical composition, and investigate its potential anticancer effects in vitro and in vivo. Specifically, we investigated the antiproliferative, antimigratory, and pro-apoptotic potential of the essential oil in vitro and assessed whether oral administration of the oil attenuated growth of colon cancer in vivo in an experimental mouse tumor model.

## 2. Results and Discussion

### 2.1. Chemical Composition

Volatile profile analysis by GC/MS identified the composition of the extracted oil (Table 1). A total of 64 compounds representing 95.06% of the total chromatographic area were identified. Carvacrol was found to be the most abundant phytochemical (47.99%) in *Origanum onites* essential oil (OOEO). This observation is in accordance with the literature, as previous analyses concluded that carvacrol is typically the main constituent in OOEO. The oil’s content in carvacrol ranged from ≈50% to even ≈85% [20,21,22]. Other major compounds that were identified were terp-1-in-4-ol (6.79%), sabinene hydrate (6.14%), γ-terpinene (5.20%), p-cymene (3.85%), and α-terpineol (3.76%). These compounds were previously reported in the literature to be among the main constituents of OOEO [18,20,23].

### 2.2. Antiproliferative Activity of OOEO

**Figure 1:**
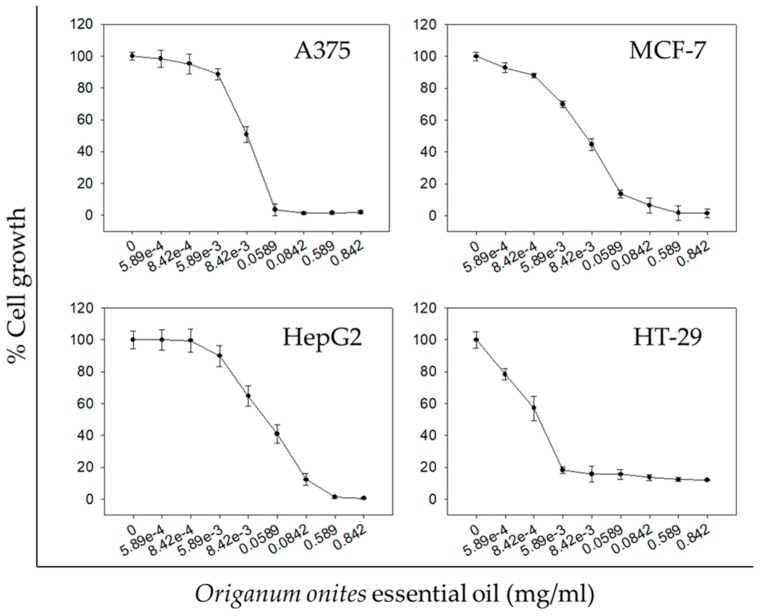
In vitro anticancer activity of OOEO against human cancer cell lines. Antiproliferative effect of increasing doses of OOEO at 72 h on human skin melanoma A375 cells, breast cancer MCF-7 cells, hepatocellular carcinoma HepG2 cells, and colon cancer HT-29 cells. Cell growth percentage was analyzed with the sulforhodamine B (SRB) assay. All data shown are representative of at least three independent experiments. Values represent mean ± SD.

OOEO was examined for its antiproliferative activity against a panel of human cancer cell lines from four different tumor types. In particular, melanoma cells (A375), breast cancer cells (MCF-7), hepatocellular carcinoma cells (HepG2), and colon cancer cells (HT-29) were treated with increasing concentrations of the essential oil for 72 h. Interestingly, OOEO exhibited a dose-dependent antiproliferative activity against all human cancer cell lines tested (Figure 1). However, the different cell lines exhibited different sensitivity to the OOEO, evident by the different IC_50_ values (Table 2). The strongest antiproliferative effect (lowest IC_50_ value for 72 h) was observed in the HT-29 colon cancer cell line (0.35 ± 0.2 μg/mL) followed by A375 skin melanoma (8.90 ± 0.7 μg/mL), MCF-7 breast carcinoma (10.0 ± 1.7 μg/mL), and HepG2 hepatocellular carcinoma cells (23.0 ± 4.2 μg/mL). 

Even though the antiproliferative potential of OOEO has not been extensively studied, the few relevant studies conclude that the oil exhibits potent and interesting growth inhibitory effects against certain cancer cell lines. Specifically, OOEO has proven to inhibit the in vitro proliferation and induce apoptosis in 5RP7 cells (c-H-ras transformed rat embryonic fibroblasts) in a concentration and a time-dependent manner [17]. Significantly, the antiproliferative effects of OOEO against HepG2 cells were also previously reported by Özkan and Erdoǧan [18], though for a different concentration range and a shorter treatment period as compared to the results reported here. Özkan and Erdoǧan also concluded that OOEO exhibited concentration- and time-dependent growth inhibitory activity against HepG2 cells. Finally, in another study, the growth of human glioblastoma (U87) and breast cancer (MDA-MB231) cells was found to be inhibited after treatment with ethanolic extracts from the *Origanum onites* plant [19].

**Figure 2:**
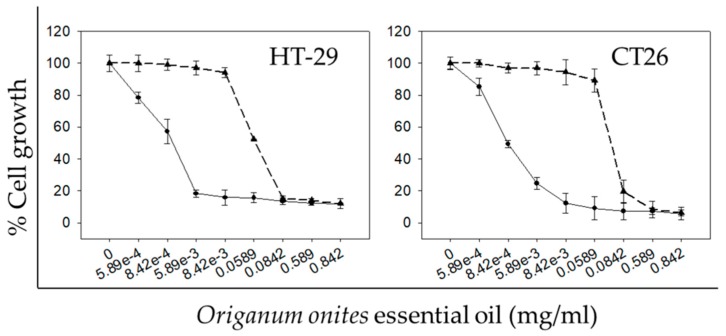
Time-dependent in vitro anticancer activity of OOEO against colon cancer cell lines. Antiproliferative effect of increasing doses of OOEO at 72 h (solid lines, circles) or 48 h (dashed lines, triangles) on human (HT-29) and mouse (CT26) colon cancer cells. Cell growth percentage was analyzed with the SRB assay. All data shown are representative of at least three independent experiments. Values represent mean ± SD.

Having observed a significant concentration-dependent growth inhibitory effect exhibited by OOEO in the human colon cancer cell line HT-29 (Figure 1), we proceeded to assay the CT26 murine colon cancer cell line as well. Moreover, we investigated whether the observed antiproliferative effect was also time-dependent. OOEO inhibited both CT26 and HT-29 cell growth in a concentration- and a time-dependent manner, evident by the concentration-effect curves (Figure 2) and the smaller IC_50_ values for the 72 h compared to the 48 h treatment, respectively (Table 2 and Table 3). Specifically, the mean IC_50_ values for the 72 h-treatment were determined to be 0.35 and 1.10 μg/mL compared to 58.00 and 71.70 μg/mL for the 48 h-treatment for HT-29 or CT26 cells, respectively. Interestingly, for both 48 and 72 h, human HT-29 cells exhibiting smaller IC_50_ values seemed to be more sensitive to OOEO than murine CT26 cells.

Despite the lack of previous studies regarding the antiproliferative effects of OOEO in colon cancer cells, there are certain papers reporting the growth inhibitory potential of extracts of other *Origanum* species against colorectal cancer. The ethanolic extract from *Origanum vulgare* has been shown to induce apoptosis in Caco-2 human colon cancer cells [24], while the methanolic extract has been reported to inhibit HCT-116 human colon cancer cells [25]. Moreover, the essential oil extracted from the same plant has been reported to inhibit the proliferation of HT-29 cells [26]. The aqueous extract and certain organic fractions from *Origanum marjorana* were also reported to inhibit the growth of HT-29 cells [27]. Lastly, the phenolic monoterpenoid carvacrol, which was identified as the major constituent in OOEO (47.99%) (Table 1), also possesses significant antiproliferative properties against colon cancer cells, as it was reported to induce cell cycle arrest and apoptosis in two human colon cancer cell lines, HCT-116 and LoVo [28]. Moreover, carvacrol’s antiproliferative potency has been studied against various in vitro cancer models, including the four cell lines that were used in the present study [29,30,31,32,33], suggesting that OOEO’s observed antiproliferative activity could be attributed to its high content in carvacrol. In addition, there are studies that indicate that, among the phytochemicals present in essential oils, there is a strong synergistic/additive effect for cancer cell growth inhibition [34,35]. Carvacrol has specifically been reported to act synergistically with other compounds present in OOEO for exerting cytotoxicity against various parasitic organisms [36,37]. There are no published data, however, regarding carvacrol’s potential synergistic effects with other phytochemicals against cancer cell lines. Thus, we suggest that future research should be focused on the investigation of whether OOEO’s antiproliferative activity against colon cancer cells can be attributed to its main constituents and on determining whether there are synergistic interactions among them.

### 2.3. OOEO Attenuates Migration of Colon Cancer Cells In Vitro

**Figure 3:**
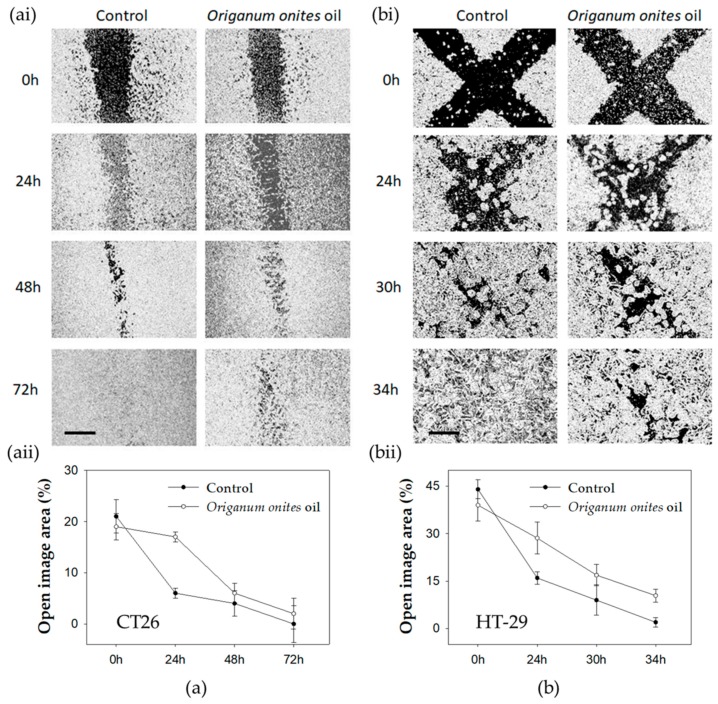
Effect of OOEO on migration of colon cancer cells. Wound-healing assay for (**a**) CT26 and (**b**) HT-29 cells treated with the oil (0.70 μg/mL for CT26 and 0.50 μg/mL for HT-29 cells) or dimethylsulfoxide (DMSO) for control. Migration of (**ai**) CT26 (scale bar = 500 μm) and (**bi**) HT-29 cells (scale bar = 300 μm) was monitored with an optical microscope equipped with a digital camera at the indicated time points. Quantification of the percentage of wound closure was estimated by ImageJ software analysis for (**aii**) CT26 and (**bii**) HT-29 cells. Data are presented as the mean ± SD of three independent experiments.

The ability of cancer cells to migrate is essential for tumor invasion and metastasis [38]. Therefore, we investigated whether OOEO could attenuate HT-29 or CT26 colon cancer cells migration rate in vitro with the wound healing assay. Our results indicate that in both HT-29 and CT26 cells, the open area (wound) closed earlier in control cells compared to the cells that were treated with low, non-toxic concentrations of OOEO (Figure 3). For CT26 cells, in the control group, migration was very dynamic in the early stages of the experiment, as the mean ratio of the open area reached 6% after only 24 h from the initial 21% in 0 h, while the treated group reached the same percentage of closure after 48 h. Wound closure occurred after 72 h in control cells, while the OOEO-treated cells exhibited a remaining 2–5% of open image area for the same time point (Figure 3aii). The control group for HT-29 cells reached a 0–2% of open image area (complete wound closure) in 34 h, while in the OOEO-treated cells, the wound closure was not complete in 34 h, as the mean open area was at 10% (Figure 3bii). 

There are no other reports to our knowledge regarding the OOEO-induced inhibition of cancer cells migration. However, Bostancıoğlu et al. described the antimigratory effect of OOEO in RATEC cells (rat adipose tissue endothelial cells) [17]. Nevertheless, there are certain reports on the antimigratory effects exerted in cancer cells by extracts of different *Origanum* species, such as the ethanolic or the methanolic extract of *Origanum syriacum* against LoVo and SW620 human colon cancer cell lines [39] or against the MDA-MB-231 human breast cancer cell line [40], respectively. *Origanum marjorana* ethanolic extract has also been reported to attenuate the migration rate of the highly metastatic MDA-MB-231 cancer cell line [41]. Similarly to OOEO’s antiproliferative effects described in 2.2, its antimigratory activity could also be partly attributed to its main constituent, carvacrol, as carvacrol has been reported to inhibit the migration of various human cancer cells in vitro, such as HCT-116 and LoVo colon cancer cells [28], non-small cell lung cancer (NSCLC) cells [42], oral squamous cell carcinoma Tca-8113 and SCC-25 cells [43], and PC3 prostate cancer cells [44].

### 2.4. OOEO Induces Apoptosis-Related Morphological Changes in Colon Cancer Cells In Vitro

**Figure 4:**
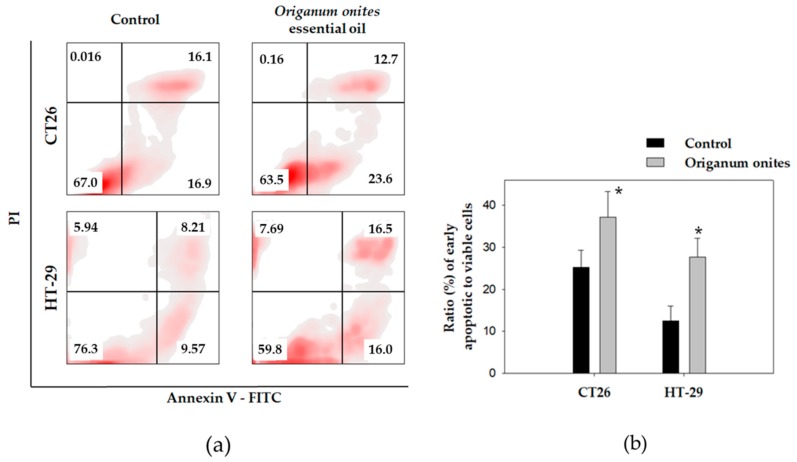
OOEO induces apoptotic cell death in colon cancer cells detected by flow cytometry. CT26 or HT-29 cells were treated with 71.70 or 58.00 μg/mL of the oil, respectively, for 24 h. Apoptotic cell death of cells was detected by dual staining with Annexin V-FITC and PI followed by flow cytometric analysis. (**a**) The percentages of the following cell populations are indicated on the respective density plots: Annexin V-FITC and PI negative stained, indicating viable cells (lower left quadrant), Annexin V-positive and PI-negative stained, indicating early apoptotic cells (lower right quadrant), and Annexin V/PI double-stained cells, showing late apoptosis (upper right quadrant). (**b**) Ratio percentage of early apoptotic to viable cells for each cell line. At least 10,000 cells were analyzed per sample. Results are presented as the mean ± SD of three independent experiments. Asterisks indicate statistically significant difference between control and treated cells (*p* < 0.05, Student’s t-test).

Since extracts from different *Onites* species as well as carvacrol have been reported to induce apoptotic cell death in different colon cancer cell lines in vitro [24,28], the pro-apoptotic potential of OOEO against the colon cancer cell lines studied here was investigated by the flow cytometric analysis of Annexin V and propidium iodide (PI) double-stained OOEO-treated cells. Our results indicate that OOEO induced morphological changes typically observed in apoptosis in both CT26 and HT-29 cells after treatment with 71.70 or 58.00 μg/mL, respectively, for 24 h (Figure 4). For CT26, the percentage ratio of early apoptotic to viable cells increased from 25 ± 4% in the control to 37 ± 6% in the OOEO-treated cells (*p* = 0.04, Student’s t-test), while treatment of HT-29 cells with OOEO induced a rise in the pro-apoptotic fraction of cells from 12 ± 3% in the control group to 28 ± 5% (*p* = 0.01, Student’s t-test) (Figure 4b). Apoptosis is a very crucial process in cancer development and a very popular target of new anticancer strategies [45]. Natural products in general are a very valuable source of new anticancer compounds [46]. Essential oils from certain plants that are mixtures of several bioactive compounds can be the basis for the identification of novel anticancer molecules that can further be exploited in therapeutics [47,48]. Our results indicate that OOEO’s antiproliferative activity against CT26 and HT-29 colon cancer cells could be partially attributed to apoptosis-related mechanisms, which makes OOEO a great candidate for further studies.

### 2.5. Oral Administration of OOEO Inhibits In Vivo Growth of Colon Carcinoma in Mice

**Figure 5:**
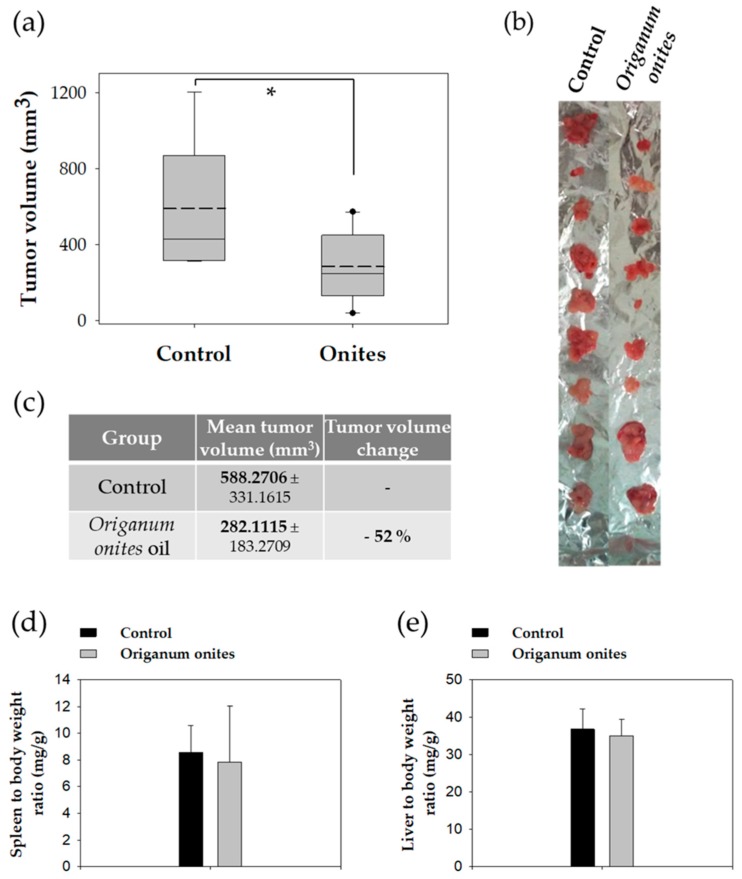
Oral administration of OOEO inhibits the growth of colon carcinoma in mice. OOEO was administered orally daily to BALB/c mice for 13 days (0.370 g/kg body weight/day). On the 10th day, mice were inoculated subcutaneously with CT26 cancer cells, and seven days later, tumors were harvested from euthanized animals (*n* = 10 per group). A statistically significant reduction of ≈52% in tumor volume (*p* = 0.022, Student’s t-test) was observed in the group that was receiving the oil as compared to the control mice. (**a**) Graphical representation of tumor volume distribution (solid lines indicate median and dashed lines indicate mean values), (**b**) photographic observation, and (**c**) mean tumor volume of excised tumors. No significant differences were observed between (**d**) spleen or (**e**) liver weight to body weight ratios between control and oil-treated mice. Asterisk indicates a statistically significant difference.

Oral administration of OOEO (0.370 g/kg body weight/day) for 13 days significantly inhibited CT26-tumor growth in BALB/c mice compared to control animals that were receiving corn oil (vehicle) in the same dosage scheme (Figure 5). Tumors from OOEO-treated animals had a significantly lower mean tumor value by 52% (*p* = 0.022, Student’s t-test) (Figure 5c). Notably, during the experimental procedure, no signs of disease or discomfort (including body weight change) were observed in either mice group. Moreover, there were no significant differences between control and OOEO-treated mice regarding mean spleen or mean liver weight to total body weight ratios (Figure 5d,e), which are sensitive indicators for the detection of toxic effects in BALB/c mice [49].

As far as we know, this is the first report on the antitumor potential of orally administered OOEO. Crucially, even though significant findings have been reported in the literature that indicate the anticancer activity of essential oils extracted from plants of the *Origanum* species [50], in vivo studies are scarce. Only the essential oil from *Origanum vulgare* L. has been assayed on an in vivo tumor model. Specifically, Misharina et al. reported that oral administration of the *Origanum vulgare* oil in F1 DBA C57 black hybrid mice inhibited the formation and the growth of engrafted Lewis carcinoma tumors [51]. Interestingly, the aqueous extract of the same plant was also shown to exert a dose-dependent anticarcinogenic effect on DMH-induced tumor formation in Wistar rats [52]. Nevertheless, our results highlight the importance of further studying *Origanum* essential oils using in vivo tumor models, and, specifically, the importance of examining the in vivo anticancer potency of OOEO with various short and long term dosage schemes and in various experimental models, such as models for carcinogenesis or metastasis.

It is crucial to note that the 52% inhibition of tumor growth observed in the present study was achieved with the short term (13 days) daily oral administration of 370 mg/kg of body weight OOEO, a dosage scheme that did not induce toxic effects. Unfortunately, there are no safety assessment reports on OOEO. However, Cabello et al, who tested the essential oil extracted from *Origanum vulgare* L. *virens*, which shares the same major constituents with OOEO such as carvacrol, p-cymene, terpinen-4-ol, α-terpinene, and γ-terpinene, concluded on the safety of the daily oral administration of 200 mg/kg of body weight (about half of the dose used in the present study) in Wistar rats for 90 days [53]. Notably, the duration of the administration scheme followed in the present study was much shorter (six times less). 

These results provide compelling in vivo evidence for the potential safety and the efficacy of orally administrated OOEO against colorectal cancer.

## 3. Materials and Methods 

### 3.1. Plant Material 

Air-dried herb material of *Origanum onites* was obtained from the company Alexopoulos Alexandros & Co (Athens, Greece). The herb was collected during the maximum blooming period from regions of northern Greece, dried, and screened by hand. Only the leaves and the flowers were used for the extraction of the essential oil. The dried herb was ground in a laboratory mill (Retch ZM 1; Haan, Germany) equipped with a 0.5 mm sieve.

### 3.2. Essential Oil Extraction

The herb was subjected to hydrodistillation in a laboratory scale water-steam distillation apparatus for 6 h. A batch of 200 g was mixed with 2.5 L of water, and steam was supplied through the mixture. The collected essential oil was dried over anhydrous sodium sulphate and kept in a sealed glass vial at 4 °C. The yield of the essential oil obtained by hydrodistillation of the dried herb was 2.47% ± 0.14 v/m, dry basis.

### 3.3. Gas Chromatography and Mass Spectroscopic Analysis (GC/MS) Analysis

GC/MS analysis of OOEO was performed in a GC-MS (GC: 6890 A, Agilent Technologies, Santa Clara, CA, USA; MSD: 5973, Agilent Technologies) using an HP-1 ms column (25 m, 0.2 mm i.d., 0.33 μm film thickness). The essential oil (0.1 μL) was directly injected, and a 1:100 split ratio was applied. The oven temperature was set at 50 °C for 1 min, followed by a temperature gradient of 2.5 °C/min. When temperature reached 160 °C, it was kept steady for 20 min. Then, a step of 5.0 °C/min was applied until oven temperature was 250 °C, where it was kept for 15 min. Helium was used as carrier gas with a flow rate of 1 mL/min. Injector and transfer line temperatures were both set to 250 °C. The mass spectrometer operated in the electron impact mode with the electron energy set to 70 eV. Volatiles identification was completed according to the standard method of Kováts Indices and mass spectra comparison to Willey/NIST 0.5 and in-house created libraries (VIORYL S.A., Chemical & Agricultural Industry, Research, Athens, Greece).

### 3.4. Chemicals and Reagents

Dulbecco’s Modified Eagle’s Medium (DMEM), DMEM high glucose, and RPMI media were purchased from Gibco^®^ (Gaithersburg, MD, USA). Fetal bovine serum (FBS), trypsin, penicillin/streptomycin, and phosphate buffered saline (PBS) were purchased from Biosera (Boussens, France). DMSO, acetic acid, TCA, Trizma base, and sulforhodamine B (SRB) were purchased from Sigma-Aldrich (Steinheim, Germany). Annexin V-PI kit was purchased from BD Biosciences (San Jose, CA, USA).

### 3.5. Cell Lines

All cell lines were maintained under sterile conditions at 37 °C in a humidified atmosphere of 5% CO_2_ and routinely passaged with trypsin. Human HT-29 and murine CT26 colon carcinoma cell lines were cultured in DMEM. Human MCF-7 (breast adenocarcinoma) and HepG2 (hepatocellular carcinoma) were also maintained in DMEM, while human A375 cells (malignant melanoma) were cultured in DMEM high glucose. All media were supplemented with 10% fetal bovine serum, penicillin (100 U/mL), and streptomycin (100 μg/mL).

### 3.6. Cell Proliferation Assay

Cell proliferation was determined by the Sulforhodamine B (SRB) assay [54] as previously described [34]. Briefly, cells were seeded in 96-well plates and treated with increasing concentrations of OOEO dissolved in DMSO (1:1 *v*/*v*) for 48 h or 72 h before being fixed with TCA and stained with SRB dissolved in acetic acid. Control cells were incubated in DMSO-containing DMEM (DMSO concentration ≤ 0.1% *v*/*v*). The cell-bound SRB dye was dissolved in Tris base, and the sample’s absorbance (optical density, OD) was measured at 492 nm using a microplate reader (Enspire, Perkin Elmer, Waltham, MA, USA). OD values are indicative of the amount of dye extracted from stained cells, which is proportional to the cell mass. The IC_50_ values (efficient concentration that causes a 50% decrease in cell viability) were calculated by regression analysis conducted using the Sigma Plot Software (v.11, Systat Software Inc., San José, CA, USA). At least six replicates for each sample were examined, and each experiment was independently performed at least three times. The percentage of inhibition of cell growth was calculated by the following formula:

% *growth* = *mean OD sample*/*mean OD control* × *100* (1)

### 3.7. Wound Healing Assay

In order to investigate the antimigratory potential of OOEO, we employed the wound healing assay. CT26 cells were seeded in 35 mm culture dishes with IBIDI silicon inserts (Cat.No: 80209, IBIDI Gräfelfing, Germany) consisting of two reservoirs separated by a 500 μm wall, while HT-29 cells were seeded in regular 12-well plates. After an overnight incubation, the IBIDI inserts were removed from the CT26-seeded dishes, creating a 500 μm wide wound (Figure 3ai). Moreover, a 200 μL pipette tip was used to scratch the monolayer formed by HT-29 cells across the well twice, thus a cross was formed from the resulting gaps (Figure 3bi). In order to exclude the possibility that the wound healing process was attenuated due to the growth inhibitory effects of OOEO, we used non-toxic concentrations for the treatment of the cells that did not inhibit cell growth. Thus, CT26 cells were treated with 0.70 μg/mL of OOEO, while HT-29 cells were treated with 0.50 μg/mL. Control cells were treated with DMSO (DMSO concentration ≤ 0.1% *v*/*v*). Cells were photographed at indicated time points with a ZEISS Primovert light microscope (Zeiss, Göttingen, Germany) equipped with a digital camera (Axiocam ERc 5 s). Multiple photographs per time point were analyzed with ImageJ software (NIH, Bethesda, MD, USA), and the average wound area percentage (percentage of open image area) was calculated for both cell lines.

### 3.8. Flow Cytometric Analysis of Apoptosis by Annexin V and Propidium Iodide Staining

Apoptosis was assayed using the Annexin V-PI method [55]. A commercially available kit was used according to the manufacturer’s instructions. CT26 or HT-29 cells were seeded in 10 cm^2^ cell culture plates and treated with 71.70 or 58.00 μg/mL of OOEO, respectively (IC_50_ values for 48 h) for 24 h. Untreated cells were used as the control. Following treatment, cells were collected and handled according to the manufacturer’s protocol. After being subsequently labeled with Annexin V-FITC and PI, cells were analyzed on a flow cytometer (Attune NxT, Thermo Fisher Scientific, Waltham, MA, USA). Data analysis was performed with FlowJo V10 software (Tree Star, Inc., Ashland, OR, USA). 

### 3.9. Animals

Twenty female BALB/c mice (6–8 weeks old, weight 20–25 g) were purchased from the Animal Facility of Pasteur Institute (Athens, Greece) and kept in the Animal House of Medical School at the University of Ioannina (Greece), where they were housed in polycarbonate cages. A maximum of 5 mice were housed per cage. Mice were maintained at room temperature on a 12 h light-12 h dark cycle and were provided with tap water ad libitum and a commercial pelleted diet (Mucedola). The experimental protocol followed in the present study was approved by the Animal Care and Use Committee of the Veterinary Service in Ioannina and was in compliance with Directive 86/609/EEC. 

### 3.10. CT26 Experimental Tumor Model

Mice were separated into two independent groups (10 mice per group), and OOEO was administered orally in a final volume of 100 μL at a daily dose of 0.370 g/kg of animal body weight for 13 days. Administration of OOEO was performed by oral gavage. Mice in the control group received an equal volume of corn oil (vehicle). Animals were weighted daily and monitored for signs of disease or discomfort. At day 10, 5 × 10^6^ cultured CT26 cells were subcutaneously injected on the scruff of the neck of all animals; 7 days later, mice were euthanized by cervical dislocation, and tumors were harvested. Moreover, the spleen and the liver were surgically removed and weighted. Tumor dimensions were defined by an electronic micrometer, and tumor volume (mm^3^) was calculated by the modified ellipsoid formula:

0.5 × (width^2^ × length) (2)

## 4. Conclusions

In conclusion, the present study provides novel evidence on antiproliferative, antimigratory, and pro-apoptotic potency of the essential oil extracted from *Origanum onites* against colon cancer. After investigating the growth inhibitory effects of the oil against a panel of four different cancer cell lines representing different tumor types, we concluded that, among the cell lines studied, colon cancer cells were the most sensitive to OOEO’s antiproliferative activity. Moreover, when administered orally, OOEO inhibited the growth of colon carcinoma tumors in mice. Our results suggest that OOEO could be used as a dietary nutraceutical for colon cancer prevention. Considering the lack of relevant in vivo studies regarding the potential anticancer activity of essential oils from *Origanum* species and based on our findings, we strongly believe that future work should concentrate on the in vivo evaluation of the antitumor potential of the oils and their constituents. Regarding OOEO in particular, we are currently in the process of characterizing the underlying molecular mechanisms involved in its antiproliferative activity along with evaluating its safety and its anticancer activity in various dosage/exposure schemes in explant cultures of human, normal, and cancerous colon tissues.

## Figures and Tables

**Table 1:** Volatile compounds identified in *Origanum onites* essential oil (OOEO) by GC/MS analysis and their relative percent (%) area.

KRI *	Compounds	% Area
765	methyl 2-methylbutyrate	0.006
845	isoamyl acetate	0.022
846	2-methyl butyl acetate	0.005
921	thujene	0.336
927	α-pinene	0.255
939	camphene	0.186
939	ethyl amyl ketone	0.093
939	sabinene	0.094
964	β-pinene	0.117
977	myrcene	0.803
990	α-phellandrene	0.161
998	3-carene	0,028
1003	α-terpinene	2.257
1007	p-cymene	3.847
1014	β-phellandrene	0.45
1015	limonene	0.371
1022	ocimene ( trans )	0.041
1034	ocimene ( cis )	0.094
1047	γ-terpinene	5.206
1050	thujanol	1.277
1054	epoxy linalool (cis)	0.020
1069	epoxy linalool (trans)	0.013
1072	Dehydro-p-cymene	0.016
1077	terpinolene	0.813
1084	sabinene hydrate	6.135
1086	linalool	2.048
1095	oct-1-en-3-yl acetate	0.051
1107	p-2-menthen-1-ol (cis)	0.748
1110	oct-3-yl acetate	0.02
1119	camphor	trace
1123	p-2-menthen-1-ol (trans)	0.479
1150	borneol	1.97
1163	terp-1-in-4-ol	6.786
1173	α-terpineol	3.756
1189	piperitol (cis)	0.136
1194	piperitol (trans)	0.147
1216	nerol	0.092
1220	carvone	0.152
1220	citral (cis)	0.005
1221	isopiperitenone	trace
1226	carvacrol methyl ether	0.079
1240	linalyl acetate	1.204
1243	citral (trans)	0.464
1262	indol	0.048
1279	thymol	0.428
1296	carvacrol	47.988
1347	neryl acetate	0.179
1350	thymyl acetate	0.001
1365	geranyl acetate	0.336
1416	caryophyllene	1.167
1423	thymohydroquinone	0.621
1436	aromadendrene	0.125
1449	humulene	0.059
1474	germacrene D	0.106
1491	bicyclogermacrene	0.251
1402	β-bisabolene	0.556
1406	γ-cadinene	0.066
1416	δ-cadinene	0,052
1464	spathulenol	0.393
1469	caryophyllene oxide	0.327
1525	cadinol	0.239
1964	unknown	0.968
2031	ar-abietatriene	0.254
2294	4-epi-dehydroabietol	0.112

* KRI: Kovats Retention Indices.

**Table 2:** IC_50_ values (efficient concentration that causes a 50% decrease in cell growth) of OOEO against different cancer cell lines determined with the SRB assay after a 72 h treatment. Data are representative of at least three independent experiments and are presented as mean ± SD.

Cell Line	IC_50_ (72h)
**A375**	8.90 ± 0.70 μg/mL
**MCF-7**	10.00 ± 1.70 μg/mL
**HepG2**	23.00 ± 4.20 μg/mL
**HT-29**	0.35 ± 0.20 μg/mL
**CT26**	1.10 ± 0.30 μg/mL

**Table 3:** IC_50_ values (efficient concentration that causes a 50% decrease in cell growth) of OOEO against human (HT-29) and mouse (CT26) colon cancer cells determined with the SRB assay after a 48 h treatment. Data are representative of at least three independent experiments and are presented as mean ± SD.

Cell Line	IC_50_ (48h)
**HT-29**	58.00 ± 0.70 μg/mL
**CT26**	71.70 ± 1.20 μg/mL
